# Supplementary material for: Yeast Volatomes Differentially Affect Larval Feeding in an Insect Herbivore
Source: Appl Environ Microbiol. 2019 Oct 16;85(21):e01761-19. doi: 10.1128/AEM.01761-19 (PMC6803314; doi:10.1128/AEM.01761-19)
Supplement: Supplemental file 1 [file AEM.01761-19-s0001.pdf]

TABLE S1 Volatile compounds identified by GC-MS from the headspace of eight yeasts, *Cryptococcus nemorosus*, *M. andauensis*, *M. fructicola*, *M. hawaiiensis*, *M. lopburiensis*, *M. pulcherrima*, *M. saccharicola* and *Saccharomyces cerevisiae*. Compounds marked with a bullet are not listed in yeast databases, two bullets for compounds not known from databases of yeast, bacterial or fungal metabolites. Compound nomenclature follows IUPAC, according to PubChem. CAS and CID number are shown, and Kovats indices on a DB-Wax column. Letters R and W denote compounds listed in yeast metabolome databases by Ramirez-Gaona et al. (2017)[a] and Weldegergis et al. (2011)[b], followed by the number of bacteria and fungi, respectively, producing these compounds according to Lemfack et al. (2018)[c].

	Compound	Synonym	CAS number	PubChem CID	Kovats DB-Wax	Database records[a,b,c]
	Ethyl acetate		141-78-6	8857	800	R,W,10,30
•	Methanol		67-56-1	887	812	12,1
	Ethanol		64-17-5	702	884	R,18,42
	1,1-Diethoxyethane		105-57-7	7765	890	R,W,0,0
	Methyl 2-methylpropanoate		547-63-7	11039	920	R,12,4
	Ethyl propanoate		105-37-3	7749	954	R,W,1,13
	Ethyl 2-methylpropanoate		97-62-1	7342	963	W,3,2
	Propyl acetate		109-60-4	7997	971	R,W,1,2
	Methyl butanoate		623-42-7	12180	982	R,W,14,8
	1-(1-Ethoxyethoxy)butane		57006-87-8	552119	997	W,0,0
•	(±)-Methyl 2-methylbutanoate		868-57-5	13357	1007	13,9
	2-Methylpropyl acetate		110-19-0	8038	1010	R,W,3,2
•	Methyl 3-methylbutanoate		556-24-1	11160	1016	28,2
	Ethyl butanoate		105-54-4	7762	1034	R,W,10,8
	(±)-Ethyl 2-methylbutanoate		7452-79-1	24020	1051	R,W,16,13
	Ethyl 3-methylbutanoate		108-64-5	7945	1067	R,W,5,6
	2,2-Dimethyl-3-methylidenebicyclo[2.2.1]heptane	Camphene	79-92-5	6616	1067	R,1,18
	Butyl acetate		123-86-4	31272	1070	R,W,9,3
	2-Methyl-propan-1-ol	Isobutanol	78-83-1	6560	1078	R,47,52
•	2-Methylpropyl 2-methylpropanoate		97-85-8	7351	1090	0,3
•	(±)-Methyl 2-methylpentanoate		2177-77-7	519890	1096	2,0
	(±)-2-Methylbutyl acetate		624-41-9	12209	1113	R,W,2,2
	3-Methylbutyl acetate		123-92-2	31276	1121	R,W,12,7
•	Heptan-4-one		123-19-3	31246	1125	8,1
••	(±)-Methyl 3-methylpentanoate		2177-78-8	519891	1129	0,0
•	Heptane-2,3-dione		96-04-8	60983	1146	1,0
	Heptan-3-one		106-35-4	7802	1153	W,1,0
	2-Metylpropyl butanoate		539-90-2	10885	1158	R,0,0
	7-Methyl-3-methylideneocta-1,6-diene	β-Myrcene	123-35-3	31253	1162	R,W,2,9
	Pentyl acetate		628-63-7	12348	1171	R,W,0,1
	Heptan-2-one		110-43-0	8051	1182	R,W,32,21
	Methyl hexanoate		106-70-7	7824	1186	R,W,2,0
	1,2-Xylene		95-47-6	7237	1186	R,4,3
	3-Methylbutyl propanoate		105-68-0	7772	1188	R,2,1
••	(±)-Hexan-3-ol		623-37-0	12178	1192	0,0
	(±)-2-Methylbutan-1-ol	Amyl alcohol	137-32-6	8723	1202	R,W,40,52
	3-Metylbutan-1-ol		123-51-3	31260	1204	R,W,128,67
	(±)-Hexan-2-ol		626-93-7	12297	1216	R,W,0,7
	Butyl butanoate		109-21-7	7983	1218	R,3,0
	2-Pentylfuran		3777-69-3	19602	1230	R,W,3,63
	Ethyl hexanoate		123-66-0	31265	1234	R,W,10,12
•	Ethyl (E)-2-methylbut-2-enoate		5837-78-5	5281163	1237	0,2
•	3-Methylbut-3-en-1-ol	Isoprenol	763-32-6	12988	1246	23,2
	Pentan-1-ol		71-41-0	6276	1246	R,4,45

	Unknown 45				1251	-
	Octan-3-one		106-68-3	246728	1255	R,8,51
•	3-Methylbutyl butanoate		106-27-4	7795	1266	2,1
	Hexyl acetate		142-92-7	8908	1271	R,W,1,0
•	Heptan-4-ol		589-55-9	11513	1279	1,0
•	(±)-2-Methylbutyl 2-methylbutanoate		2445-78-5	17129	1281	0,3
	3-Hydroxybutan-2-one	Acetoin	513-86-0	179	1285	R,69,14
••	2-Ethoxyetyl acetate		111-15-9	8095	1289	0,0
	Octanal		124-13-0	454	1289	R,W,10,3
••	Methyl (*E*)-hex-2-enoate		2396-77-2	5364409	1291	0,0
•	(±)-2-Methyl pentan-1-ol		105-30-6	7745	1295	0,1
•	Cyclohexanone		108-94-1	7967	1300	3,0
	2-Ethylbutan-1-ol		97-95-0	7358	1304	W,0,0
••	(3*E*)-4,8-dimethylnona-1,3,7-triene		19945-61-0	6427110	1306	0,0
	4-Methylpentan-1-ol	iso-Hexanol	626-89-1	12296	1310	R,W,0,1
	Heptan-2-ol		543-49-7	10976	1315	R,W,7,7
	(*Z*)-Hex-3-enyl acetate		3681-71-8	5363388	1316	R,0,0
•	2-Methylbut-2-en-1-ol		4675-87-0	20799	1317	1,0
	(±)-3-Methylpentan-1-ol		589-35-5	11508	1324	R,W,0,2
•	Nonan-4-one		4485-09-0	78236	1328	0,2
•	Ethyl 3-ethoxypropanoate		763-69-9	12989	1330	0,1
	Ethyl heptanoate		106-30-9	7797	1333	R,W,0,0
	6-Methylhept-5-en-2-one	Sulcatone	110-93-0	9862	1338	R,W,24,30
	Ethyl hex-2-enoate		1552-67-6	5364778	1346	R,0,1
	1-Hexanol		111-27-3	8103	1349	R,W,34,53
	Unknown 70				1361	-
	Unknown 71				1367	-
	3-Ethoxy-propan-1-ol		111-35-3	8109	1376	R,0,0
	(*Z*)-Hex-3-en-1-ol		928-96-1	5281167	1382	R,W,0,6
	Methyl octanoate		111-11-5	8091	1389	R,W,0,0
	Nonan-2-one		821-55-6	13187	1391	R,W,87,18
	Nonanal		124-19-6	31289	1395	R,22,37
••	(±)-Methyl 2-hydroxy-3-methylbutanoate		17417-00-4	552631	1399	0,0
••	1,3,5-Undecatriene		16356-11-9	5367412	1406	0,0
•	(*E*)-Oct-3-en-2-one		1669-44-9	5363229	1411	0,2
•	Butyl hexanoate		626-82-4	12294	1414	1,0
	(±)-4-Hydroxyhexan-3-one	Propionoin	4984-85-4	95609	1415	R,1,1
••	Hexyl butanoate		2639-63-6	17525	1416	0,0
••	(±)-Hexyl 2-methyl butanoate		10032-15-2	24838	1429	0,0
	Unknown 84				1431	-
	Unknown 85				1432	-
	Ethyl octanoate		106-32-1	7799	1436	R,W,8,2
	Unknown 87				1440	-
••	Furan-2-carbohydrazide		3326-71-4	18731	1440	0,0
	(±)-Oct-1-en-3-ol		3391-86-4	18827	1444	R,W,5,105
	Unknown 90				1445	-
**	2-Methylbutanoyl 2-methylbutanoate		1519-23-9	102642	1451	0,0
	Heptan-1-ol		111-70-6	8129	1452	R,W,37,39
	(±)-6-Methylhept-5-en-2-ol	Sulcatol	1569-60-4	20745	1458	W,2,0
	3-Methylbutyl hexanoate		2198-61-0	16617	1460	R,W,0,0
	(*Z*)-Hex-3-enyl butanoate		16491-36-4	5352438	1461	R,0,0
	Unknown 96				1462	-
	2-Pentylthiophene		4861-58-9	20995	1465	R,1,0
	Unknown 98				1473	-
••	(*Z*)-Hex-3-enyl (±)-2-methylbutanoate		53398-85-9	5365069	1474	0,0
	Unknown 100				1477	-
••	Methyl (±)-3-hydroxybutanoate		1487-49-6	15146	1482	0,0

	Ethyl furan-3-carboxylate		614-98-2	69201	1483	R,0,0
	(±)-2-Ethylhexan-1-ol		104-76-7	7720	1485	R,W,13,39
••	Methyl (±)-2-hydroxy-3-methylpentanoate		41654-19-7	521064	1495	0,0
	Decanal		112-31-2	8175	1501	R,W,25,39
	1-(Furan-2-yl)ethanone		1192-62-7	14505	1508	R,W,18,1
	Nonan-2-ol		628-99-9	12367	1514	R,W,1,0
	Methyl 3-methylsulfanylpropanoate		13532-18-8	61641	1528	R,0,0
	Unknown 109				1530	-
	Benzaldehyde		100-52-7	240	1533	R,W,61,48
	2-Methylthiolan-3-one		13679-85-1	61664	1540	R,W,2,0
	(±)-3,7-Dimethylocta-1,6-dien-3-ol	Linalool	78-70-6	6549	1542	R,W,5,11
	Octan-1-ol		111-87-5	957	1555	R,W,18,17
	2-Methylpropanoic acid	Isobutanoic acid	79-31-2	6590	1560	R,30,7
	Ethyl 3-methylsulfanylpropanoate	Ethyl 3-methylthio-propanoate	13327-56-5	61592	1572	R,W,0,0
	Methyl furan-2-carboxylate	Methyl 2-furoate	611-13-2	11902	1580	W,5,5
••	4-Ethylbenzene-1,3-diol		2896-60-8	17927	1584	0,0
	Unknown 118				1588	-
	Undecan-2-one		112-12-9	8163	1603	R,108,14
••	2-Methyl-1-benzofuran		4265-25-2	20263	1607	0,0
•	Benzonitrile		100-47-0	7505	1619	4,0
	Ethyl furan-2-carboxylate	Ethyl 2-furanoate	614-99-3	11980	1624	W,0,0
	Unknown 123				1628	-
	Methyl benzoate		93-58-3	7150	1634	W,6,4
	Ethyl decanoate		110-38-3	8048	1641	R,W,3,1
	2-Phenylacetaldehyde	2-Phenyl ethanal	122-78-1	998	1652	R,W,18,13
	3-Methyl butanoic acid		503-74-2	10430	1666	R,94,8
	Ethyl benzoate		93-89-0	7165	1678	R,W,0,2
•	1-Methoxy-4-prop-2-enylbenzene	Estragole	140-67-0	8815	1679	0,2
••	2-Ethyl-1-benzofuran		3131-63-3	76582	1692	0,0
	Ethyl dec-9-enoate	Ethyl 9-decenoate	67233-91-4	522255	1692	R,W,0,0
	Unknown 132				1697	-
	(±)-2-(4-methylcyclohex-3-en-1-yl)propan-2-ol	α-Terpineol	98-55-5	17100	1704	R,W,15,5
	3-Methylsulfanylpropan-1-ol		505-10-2	10448	1722	R,W,27,3
	(3*Z*,6*E*)-3,7,11-Trimethyldodeca-1,3,6,10-tetraene	(*Z*,*E*)-α-Farnesene	26560-14-5	5362889	1726	W,0,4
	Unknown 136				1731	-
	Unknown 137				1738	-
	Unknown 138				1741	-
	(3*E*,6*E*)-3,7,11-Trimethyldodeca-1,3,6,10-tetraene	(*E*,*E*)-α-Farnesene	502-61-4	5281516	1750	R,0,4
	(±)-3,7-Dimethyloct-6-en-1-ol	β-Citronellol	106-22-9	8842	1763	R,W,0,3
	Methyl 2-phenylacetate		101-41-7	7559	1768	R,2,4
	Unknown 142: sesquiterpene				1771	-
	Unknown 143: sesquiterpene				1784	-
	7-Methyl-3-methylideneoct-6-en-1-ol	γ-Isogeraniol	13066-51-8	518689	1786	W,0,0
	Ethyl 2-phenylacetate		101-97-3	7590	1793	W,0,0
	(2*Z*)-3,7-Dimethylocta-2,6-dien-1-ol	Nerol	106-25-2	643820	1801	R,W,0,0
	Unknown 147				1804	-
••	Ethyl 3-methylbenzoate		120-33-2	67117	1806	0,0
	(3*Z*)-3,7-Dimethylocta-3,6-dien-1-ol	Isogeraniol	5944-20-7	6536347	1810	W,0,0
•	Tridecan-2-one		593-08-8	11622	1815	54,1
	Unknown 151: sesquiterpene				1822	-
	2-Phenylethyl acetate		103-45-7	7654	1826	R,W,5
••	3-Pentyl-2H-furan-5-one		62527-72-4	12320141	1828	0,0
	Unknown 154				1835	-
	(2*E*)-3,7-Dimethylocta-2,6-dien-1-ol	Geraniol	106-24-1	637566	1845	R,5,1
••	Ethyl (2*E*,4*Z*)-deca-2,4-dienoate	Pear ester	3025-30-7	5281162	1847	0,0
	(5*E*)-6,10-dimethylundeca-5,9-dien-2-one	Geranyl acetone	689-67-8	1549778	1860	R,14,6

	Unknown 158				1878	-
	Unknown 159				1883	-
	Unknown 160				1888	-
	Unknown 161				1901	-
	Unknown 162				1901	-
	2-Phenylethanol		60-12-8	6054	1921	R,W,134,45
••	(5*E*)-6,10-Dimethylundeca-5,9-dien-2-ol	Fuscumol	53837-34-6	5370125	1953	0,0
	Unknown 165				1965	-
••	(3-Methylphenyl)methanol		587-03-1	11476	1969	0,0
	Unknown 167				1972	-
	Unknown 168				1979	-
	Unknown 169				2001	-
	Unknown 170				2032	-
	(±)-(6*E*)-3,7,11-Trimethyldodeca-1,6,10-trien-3-ol	trans-Nerolidol	7212-44-4	5284507	2037	R,W,1,8
	Unknown 172				2043	-
	5-Pentyloxolan-2-one	γ-Nonalactone	104-61-0	7710	2049	R,W,5,0
	Unknown 174				2056	-
	Unknown 175				2065	-
	Unknown 176				2079	-
	Unknown 177				2087	-
	Unknown 178				2089	-
	Unknown 179				2097	-
	Unknown 180				2135	-
	5-Hexyloxolan-2-one	γ–Decalactone	706-14-9	12813	2166	R,W,5,0
	Methyl hexadecanoate	Methyl palmitate	112-39-0	8181	2223	1,1
••	(2*Z*,6*E*)-3,7,11-Trimethyldodeca-2,6,10-trienal	(Z,E)-Farnesal	4380-32-9	5365890	2235	0,0
	Ethyl hexadecanoate	Ethyl palmitate	628-97-7	12366	2266	R,W,0,1
•	(±)-3,7,11-Trimethyldodeca-6,10-dienol	Dihydrofarnesol	37519-97-4	5280341	2278	0,1
••	(2*E*,6*E*)-3,7,11-Trimethyl-2,6,10-dodecatrienal	(E,E)-Farnesal	19317-11-4	5280598	2292	0,0
	5-Heptyloxolan-2-one	γ-Undecalactone	104-67-6	7714	2298	R,4,0
	Ethyl (*E*)-hexadec-9-enoate	Ethyl palmitoleate	54546-22-4	5364759	2302	R,0,0
••	(2*Z*,6*E*)-3,7,11-Trimethyl-2,6,10-dodecatrien-1-ol	(Z,E)-Farnesol	3790-71-4	1549108	2342	0,0
	(2*E*,6*E*)-3,7,11-Trimethyl-2,6,10-dodecatrien-1-ol	(E,E)-Farnesol	106-28-5	445070	2396	R,W,2,1
••	Methyl (*E*)-octadec-9-enoate	Methyl elaidate	1937-62-8	5280590	2544	0,0
	1H-Indole	Indole	120-72-9	798	2560	R,W,36,3

[a]Ramirez-Gaona M, Marcu A, Pon A, Guo AC, Sajed T, Wishart NA, Karu N, Feunang YD, Arndt D and Wishart DS. 2017. YMDB 2.0: a significantly expanded version of the yeast metabolome database. Nucleic Acids Res 45(D1):D440-D445.

[b]Weldegergis BT, Crouch AM, Górecki T, De Villiers A. 2011. Solid phase extraction in combination with comprehensive two-dimensional gas chromatography coupled to time-of-flight mass spectrometry for the detailed investigation of volatiles in South African red wines. Analytica Chimica Acta 701:98-111.

[c]Lemfack MC, Gohlke BO, Toguem SMT, Preissner S, Piechulla B, Preissner R. 2018. mVOC 2.0: a database of microbial volatiles. Nucleic Acids Res 46(D1):D1261-D1265.

TABLE S2 Class membership coefficients of model M1 for each yeast. Compounds with model coefficients (Coeff) >0.04 are shown, correlations between compounds for the entire data set were calculated as a correlation matrix and the two compounds showing the highest positive (PC) and negative correlation (NC) to each compound are tabulated.

Attractants

C. nemorosus					
**Compound**	**Coeff**	**PC**	**Compound**	**NC**	**Compound**
Ethyl (E)-2-methylbut-2-enoate	0.13	0.83	Fuscumol	-0.26	3-Metylbutan-1-ol
2-Pentylthiophene	0.12	0.57	Octan-3-one	-0.40	3-Metylbutan-1-ol
Fuscumol	0.09	0.96	Geranyl acetone	-0.25	3-Metylbutan-1-ol
Geranyl acetone	0.09	0.96	Fuscumol	-0.30	3-Metylbutan-1-ol
2-Methylpentan-1-ol	0.09	0.77	Geranyl acetone	-0.32	Acetoin
iso-Hexanol	0.07	0.73	(3E)-4,8-dimethylnona-1,3,7-triene	-0.32	3-Ethoxy-propan-1-ol
2-Methylbut-2-en-1-ol	0.06	0.72	Isoprenol	-0.31	Acetoin
Nonan-2-one	0.06	0.81	Unknown 123	-0.22	Methyl 2-hydroxy-3-methylbutanoate
Heptan-2-one	0.06	0.95	Heptan-4-one	-0.32	2-Phenylethanol
1-(Furan-2-yl)ethanone	0.05	0.86	2-Methyl-1-benzofuran	-0.34	3-Methylpentan-1-ol
Undecan-2-one	0.05	0.81	Unknown 154	-0.33	Ethyl 2-methylpropanoate
Unknown 85	0.05	0.98	Unknown 87	-0.45	3-Metylbutan-1-ol
Cyclohexanone	0.05	0.74	1-(Furan-2-yl)ethanone	-0.30	3-Metylbutan-1-ol
2-Methyl-1-benzofuran	0.05	0.86	1-(Furan-2-yl)ethanone	-0.33	2-Methylthiolan-3-one
Unknown 87	0.05	0.98	Unknown 85	-0.39	3-Metylbutan-1-ol

M. lopburiensis					
**Compound**	**Coeff**	**PC**	**Comp.**	**NC**	**Comp.**
Methanol	0.10	0.36	Unknown 154	-0.27	Ethanol
Ethyl (E)-2-methylbut-2-enoate	0.10	0.83	Fuscumol	-0.26	3-Metylbutan-1-ol
Ethyl furan-3-carboxylate	0.09	0.83	2-Ethylbutan-1-ol	-0.29	Isoprenol
Ethyl 3-ethoxypropanoate	0.08	0.75	Unknown 175	-0.21	Isoprenol
2-Ethylbutan-1-ol	0.08	0.83	Ethyl furan-3-carboxylate	-0.22	Isoprenol
Unknown 96	0.08	0.95	4-Ethylbenzene-1,3-diol	-0.34	3-Methylsulfanylpropan-1-ol
Unknown 87	0.07	0.98	Unknown 85	-0.39	3-Metylbutan-1-ol
Unknown 85	0.07	0.98	Unknown 87	-0.45	3-Metylbutan-1-ol
Ethyl 2-furanoate	0.07	0.80	Methyl 2-furoate	-0.31	Unknown 84
4-Ethylbenzene-1,3-diol	0.06	0.95	Unknown 96	-0.35	Ethanol
Acetoin	0.06	0.83	Unknown 70	-0.34	Isoprenol
Unknown 70	0.05	0.89	Propionoin	-0.37	Isoprenol
Hexyl acetate	0.05	1.00	Butyl butanoate	-0.13	Unknown 118
Unknown 177	0.05	0.93	Ethyl 3-methyltio-propanoate	-0.25	Unknown 84
2-Methylthiolan-3-one	0.05	0.91	Propionoin	-0.33	2-Methyl-1-benzofuran
Ethyl benzoate	0.04	0.67	Unknown 109	-0.25	2-Methyl-1-benzofuran
Indole	0.04	0.75	2-Methylthiolan-3-one	-0.32	2-Methyl-1-benzofuran
Benzonitrile	0.04	0.39	(Z)-Hex-3-enyl acetate	-0.21	Ethyl 2-phenylacetate
1,2-Xylene	0.04	0.78	3-Methylpentan-1-ol	-0.32	2-Pentylfuran
Ethyl palmitate	0.04	0.86	Ethyl palmitoleate	-0.25	Ethanol

M. hawaiiensis					
**Compound**	**Coeff**	**PC**	**Comp.**	**NC**	**Comp.**
Methyl 3-methylthio-propanoate	0.06	0.96	Ethyl 3-methyltio-propanoate	-0.30	Unknown 84
Ethyl 3-methyltio-propanoate	0.06	0.96	Methyl 3-methylthio-propanoate	-0.27	b-Citronellol
Methyl 2-phenylacetate	0.06	0.95	Methyl benzoate	-0.29	Unknown 84
Methyl 2-furoate	0.06	0.95	Ethyl 3-methyltio-propanoate	-0.29	Unknown 84
Methyl 2-hydroxy-3-methylbutanoate	0.06	0.99	Methyl 2-hydroxy-3-methylpentanoate	-0.29	Unknown 84
Methyl benzoate	0.06	0.95	Methyl 2-phenylacetate	-0.28	Unknown 84
Methyl 2-methylpropanoate	0.05	0.95	Methyl 2-methylbutanoate	-0.25	Sulcatol
Unknown 175	0.05	0.94	Furan-2-carbohydrazide	-0.21	Unknown 84
Unknown 177	0.04	0.93	Ethyl 3-methyltio-propanoate	-0.25	Unknown 84
Methyl 2-methylpentanoate	0.04	0.98	Methyl 3-methylpentanoate	-0.27	Unknown 84
Furan-2-carbohydrazide	0.04	0.94	Unknown 175	-0.24	Unknown 84
Methyl 3-hydroxybutanoate	0.04	0.92	Methyl 3-methylpentanoate	-0.23	Unknown 84

Repellents

M. andauensis Compound	Coeff	PC	Comp.	NC	Comp.
Isobutanoic acid	0.14	0.89	3-Methyl butanoic acid	-0.16	Unknown 87
3-Methyl butanoic acid	0.12	0.92	Heptan-4-ol	-0.26	(3E)-4,8-dimethylnona-1,3,7-triene
2-Ethylbutan-1-ol	0.10	0.83	Ethyl furan-3-carboxylate	-0.22	Isoprenol
2-Pentylthiophene	0.10	0.57	Octan-3-one	-0.40	3-Metylbutan-1-ol
Heptan-4-ol	0.08	0.92	3-Methyl butanoic acid	-0.31	(3E)-4,8-dimethylnona-1,3,7-triene
Unknown 147	0.06	0.89	Unknown 132	-0.26	Unknown 85
Unknown 132	0.05	0.89	Unknown 147	-0.27	Unknown 85
Heptan-4-one	0.04	0.96	Unknown 142: sesquiterpene	-0.26	2-Phenylethanol

S. cerevisiae Compound	Coeff	PC	Comp.	NC	Comp.
Heptan-1-ol	0.12	0.98	Ethyl octanoate	-0.22	(Z)-Hex-3-en-1-ol
Ethyl octanoate	0.11	0.99	Ethyl 9-decenoate	-0.22	2-Metylpropyl butanoate
Ethyl decanoate	0.09	1.00	Ethyl 9-decenoate	-0.19	g-Isogeraniol
Ethyl 9-decenoate	0.09	1.00	Ethyl decanoate	-0.18	g-Isogeraniol
Ethyl heptanoate	0.08	0.86	Ethyl hexanoate	-0.32	(Z)-Hex-3-en-1-ol
3-Methylbutyl hexanoate	0.08	0.69	Camphene	-0.17	Isobutanol
Ethyl hexanoate	0.06	0.86	Ethyl heptanoate	-0.29	(Z)-Hex-3-en-1-ol
Ethyl 2-phenylacetate	0.05	0.67	Unknown 175	-0.25	Unknown 84
g-Undecalactone	0.05	0.65	Methyl elaidate	-0.24	2-Methylbut-2-en-1-ol

M. pulcherrima Compound	Coeff	PC	Comp.	NC	Comp.
Sulcatone	0.13	0.99	Butyl butanoate	-0.10	Acetoin
Ethyl 3-methylbutanoate	0.12	0.64	Ethyl 3-methylbenzoate	-0.26	Unknown 96
Ethyl propanoate	0.09	0.68	Isoprenol	-0.26	Unknown 85
Nonan-2-ol	0.09	0.97	Unknown 138	-0.24	Isobutanol
Ethyl 3-methylbenzoate	0.09	0.83	(3-Methylphenyl)methanol	-0.27	Unknown 96
2- Methylbutyl acetate	0.07	0.88	3-Methylbutyl acetate	-0.28	Acetoin
2-Ethylhexan-1-ol	0.06	0.71	Linalool	-0.27	Isobutanol
Unknown 136	0.06	0.94	Unknown 161	-0.24	1-(1-Ethoxyethoxy)butane
Ethyl benzoate	0.06	0.67	Unknown 109	-0.25	2-Methyl-1-benzofuran
Amyl alcohol	0.05	0.82	Isobutanol	-0.31	Benzaldehyde
2-Methylbut-2-en-1-ol	0.05	0.72	Isoprenol	-0.31	Acetoin
Octanal	0.05	0.92	Nonanal	-0.21	Ethyl heptanoate
1,2-Xylene	0.05	0.78	3-Methylpentan-1-ol	-0.32	2-Pentylfuran
Geranyl acetone	0.05	0.96	Fuscumol	-0.30	3-Metylbutan-1-ol
Isoprenol	0.05	0.72	2-Methylbut-2-en-1-ol	-0.42	Unknown 85
Ethyl acetate	0.04	0.95	Propyl acetate	-0.26	Acetoin
Indole	0.04	0.75	2-Methylthiolan-3-one	-0.32	2-Methyl-1-benzofuran
Nonanal	0.04	0.92	Octanal	-0.27	Ethyl heptanoate

No effect

M. saccharicola Compound	Coeff	PC	Comp.	NC	Comp.
Unknown 142: sesquiterpene	0.06	0.98	Unknown 143: sesquiterpene	-0.25	2-Phenylethanol
Nonan-4-one	0.05	0.96	Heptan-4-one	-0.26	2-Phenylethanol
Heptan-4-one	0.05	0.96	Unknown 142: sesquiterpene	-0.26	2-Phenylethanol
(Z,E)-Farnesal	0.05	0.97	Unknown 169	-0.26	2-Methylbut-2-en-1-ol
Unknown 151: sesquiterpene	0.05	0.97	Unknown 160	-0.25	Isobutanol
Unknown 109	0.05	0.93	(E,E)-Farnesal	-0.27	2-Methylbut-2-en-1-ol
Unknown 162	0.04	0.92	Pear ester	-0.20	Isobutanol
Unknown 158	0.04	1.00	Unknown 160	-0.27	Isobutanol
Unknown 176	0.04	1.00	Unknown 179	-0.26	Isobutanol
Unknown 160	0.04	1.00	Unknown 158	-0.26	Isobutanol
Unknown 143: sesquiterpene	0.04	0.98	Unknown 142: sesquiterpene	-0.27	2-Phenylethanol
Unknown 167	0.04	0.88	Unknown 165	-0.27	Isobutanol

M. fructicola Compound	Coeff	PC	Comp.	NC	Comp.
2-Phenyl ethanal	0.09	0.40	Benzaldehyde	-0.21	Ethanol
3-Ethoxy-propan-1-ol	0.08	0.63	Ethyl 3-ethoxypropanoate	-0.32	iso-Hexanol
3-Metylbutan-1-ol	0.08	0.49	Isoprenol	-0.45	Unknown 85
2-Ethyl-1-benzofuran	0.06	0.84	2-Methyl-1-benzofuran	-0.32	1,2-Xylene
2-Methyl-1-benzofuran	0.06	0.86	1-(Furan-2-yl)ethanone	-0.33	2-Methylthiolan-3-one
2-Phenylethyl acetate	0.06	0.80	Sulcatol	-0.26	Heptan-2-one
3-Methylbutyl acetate	0.05	0.88	2- Methylbutyl acetate	-0.29	Acetoin
Benzaldehyde	0.05	0.60	Heptan-3-one	-0.35	Ethyl 2-methylpropanoate
g-Nonalactone	0.05	0.82	Unknown 174	-0.24	Isobutanol
Unknown 167	0.05	0.88	Unknown 165	-0.27	Isobutanol
Pentan-1-ol	0.05	0.75	(Z)-Hex-3-en-1-ol	-0.25	1,2-Xylene
1-Hexanol	0.04	0.79	g-Nonalactone	-0.29	1-(1-Ethoxyethoxy)butane
